# Caregiving burden: the impact of post intensive care syndrome

**DOI:** 10.1186/2197-425X-3-S1-A967

**Published:** 2015-10-01

**Authors:** J Torres, C Veiga, F Pinto, A Ferreira, F Sousa, R Jacinto, E Molinos, D Carvalho, C Dias, E Gomes, R Araújo

**Affiliations:** Unidade Local de Saúde de Matosinhos - Hospital Pedro Hispano, Matosinhos, Portugal; CINTESIS - Faculdade de Medicina da Universidade do Porto, Porto, Portugal

## Introduction

An increasing number of Intensive Care Unit (ICU) survivors develop psychological, cognitive and physical morbidities that may persist for years. The Post Intensive Care Syndrome (PICS) includes these multidimensional impairments and is described in the literature for patients and family.

The caregiver's role is crucial in patient's recovery after ICU, but is frequently overlooked by professionals. A few studies assessed caregiving burden, but it is still unclear how patient status (PICS) after discharge affects it.

## Objectives

The aim of our study was to evaluate caregiver's burden three months after discharge and to understand how patient PICS influenced it.

## Methods

Data from our Follow-Up Clinic at 3 months was prospectively collected from January 2013 to February 2015. “Zarit Burden Interview” was used and all caregivers who completed the questionnaire were included. Physical and non-physical components of PICS were assessed and compared with presence or absence of caregiver's burden. To evaluate the psychological components of PICS “Hospital Anxiety and Depression Scale” and “Post-Traumatic Stress Syndrome 14 Questions Inventory” were applied to patients. Demographic patient characteristics were also collected and compared with Zarit results. A *p* value of less than 0.05 was considered statistically significant.

## Results

A total of 168 caregivers completed the survey, with a response rate of 47%. Exactly half of caregivers (n = 84) reported no overburden (Zarit ≤ 21), 34.5% (n = 58) experienced low overburden (Zarit 22-40) and only 15,5% felt moderate to high overburden (Zarit ≥ 41).

When compared this results with psychological components of PICS three months after ICU, patient anxiety and depression significantly influenced the presence of caregiver burden (p = 0.03 and p = 0.008, respectively). On the other hand, when physical components were evaluated, they showed no influence in caregiver's burden. Patient gender, age, SAPS II, duration of sedation, duration of mechanical ventilation or ICU stay did not influence the burden.

## Conclusions

Recognition of factors that influence caregiver's burden is fundamental to allow early intervention. In this survey we found that non-physical dimension of PICS plays a fundamental role as a determinant of caregiving burden. Surprisingly, it's heavier for caregivers to deal daily with psychological problems than physical limitations. Despite only a minority of caregivers reported high overburden, an even better prevention and handling of PICS will probably reduce it.

